# Enhancing implementation science by applying best principles of systems science

**DOI:** 10.1186/s12961-016-0146-8

**Published:** 2016-10-04

**Authors:** Mary E. Northridge, Sara S. Metcalf

**Affiliations:** 1 Department of Epidemiology & Health Promotion, New York University College of Dentistry, 433 First Avenue, Room 726, New York, NY 10010 USA; 2 Department of Geography, The State University of New York at Buffalo, 115 Wilkeson Quad, Ellicott Complex, North Campus, Buffalo, NY 14261 USA

**Keywords:** Best principles, Complexity, Context, Implementation science, Modelling, Health equity, Oral health, Primary care, Screening at chairside, Systems science

## Abstract

**Background:**

Implementation science holds promise for better ensuring that research is translated into evidence-based policy and practice, but interventions often fail or even worsen the problems they are intended to solve due to a lack of understanding of real world structures and dynamic complexity. While systems science alone cannot possibly solve the major challenges in public health, systems-based approaches may contribute to changing the language and methods for conceptualising and acting within complex systems. The overarching goal of this paper is to improve the modelling used in dissemination and implementation research by applying best principles of systems science.

**Discussion:**

Best principles, as distinct from the more customary term ‘best practices’, are used to underscore the need to extract the core issues from the context in which they are embedded in order to better ensure that they are transferable across settings. Toward meaningfully grappling with the complex and challenging problems faced in adopting and integrating evidence-based health interventions and changing practice patterns within specific settings, we propose and illustrate four best principles derived from our systems science experience: (1) model the problem, not the system; (2) pay attention to what is important, not just what is quantifiable; (3) leverage the utility of models as boundary objects; and (4) adopt a portfolio approach to model building. To improve our mental models of the real world, system scientists have created methodologies such as system dynamics, agent-based modelling, geographic information science and social network simulation. To understand dynamic complexity, we need the ability to simulate. Otherwise, our understanding will be limited. The practice of dynamic systems modelling, as discussed herein, is the art and science of linking system structure to behaviour for the purpose of changing structure to improve behaviour. A useful computer model creates a knowledge repository and a virtual library for internally consistent exploration of alternative assumptions.

**Conclusion:**

Among the benefits of systems modelling are iterative practice, participatory potential and possibility thinking. We trust that the best principles proposed here will resonate with implementation scientists; applying them to the modelling process may abet the translation of research into effective policy and practice.

**Electronic supplementary material:**

The online version of this article (doi:10.1186/s12961-016-0146-8) contains supplementary material, which is available to authorized users.

## Background

This review is grounded in the ongoing experiences of the authors to devise and implement interventions to promote health equity, including for older adults. Because the aforementioned interventions are both multilevel and dynamic, the scientific approaches employed evolved from utilising ecological models for thinking through pathways whereby determinants at the societal, community and interpersonal levels affect population and individual health and well-being [1–4], to embracing a portfolio of systems science models that usefully inform related research, practice, policy and education initiatives [5–7].

Forrester, the founder of system dynamics, famously explained that a manager’s verbal description of a corporate organisation constitutes a model [8]. Such mental models of corporations are used by managers to deal with problems that arise on a daily basis. They are not, however, the real corporation. Rather, they substitute in our thinking for the real organisation. Sterman, a leading systems scientist modeller and extraordinary communicator, attributes the lack of learning effectively in a world of dynamic complexity to poor inquiry skills. He argues, “*We do not generate alternative explanations or control for confounding variables. Our judgments are strongly affected by the frame in which the information is presented, even when the objective information is unchanged. We suffer from overconfidence in our judgments (underestimating uncertainty), wishful thinking (assessing desired outcomes as more likely than undesired outcomes), and confirmation bias (seeking evidence consistent with our preconceptions)*” ([9], p. 510).

A complex (adaptive) system has been usefully defined as a system comprised of a large number of entities that display a high level of interactivity that is largely nonlinear, containing demonstrable feedback loops [10, 11]. The term systems science is used to refer to the ‘big picture’ of problem solving, where the problem space is conceptualised as a system of interrelated component parts [12]. Both the coherent whole of the system and the relationships among the component parts are critical to the system, as they give rise to emergence, meaning much coming from little [13]. Note that emergence occurs when even a relatively simple system generates unexpected amounts of complexity, which cannot be understood without the ability to create a model [13]. There are a number of other basic observations that have been made through the examination of complex systems, primarily through the use of computer simulation and the mathematics of nonlinearity, including self-organisation, meaning insensitive to large disturbances [14] and incompressibility, meaning any reduction in complexity will result in the loss of system aspects [15]. The overarching point is that rather than focusing on the parts of a system and how they function, one must focus on the interactions between these parts, and how these relationships determine the identity not only of the parts, but of the whole system [11].

Likewise, dissemination and implementation research places an emphasis on studying issues in context [3, 16, 17]. In his seminal article on diffusion, dissemination and implementation, Lomas explained, “*Implementation … is dependent on a complex framework of sanctions and incentives, reinforced by monitoring and adjustment, and often adapted to fit differing environments at more local levels*” ([18], p. 227). Thus, the congruence of an implementation science approach with a systems science approach is both intuitive and pragmatic. After first-hand engagement in conducting an implementation science pilot study [19, 20], however, the use of systems science modelling to strengthen the dissemination and implementation evidence base became a tangible next step rather than a future direction for the field [21].

Previous researchers have contended systems thinking may usefully advance implementation science. Indeed, Glasgow and Chambers [22] argued that implementation researchers would profit from embracing an interrelated systems perspective rather than a mechanistic, determinism approach to science. Further, Holmes et al. [23] sought to draw attention to certain implications inherent in adopting a systems view for dissemination and implementation research, especially with regard to causation and leverage points for change in a complex system. Recently, Burke et al. [24] presented case examples of three systems science methods, namely system dynamics, agent-based modelling and network analysis, to illustrate how each method may be used to address dissemination and implementation challenges. Finally, Valente conducted a review of network interventions without specifically relating them to implementation science, yet concluded that the choice of intervention depends, in part, on the social context of the program [25], in concert with the systems perspective that context is critical [22].

While complex systems science alone cannot possibly solve the major challenges in public health, it has been argued that systems-based approaches may contribute to changing the language and methods for conceptualising and acting within complex systems [26]. Moreover, it may eventually improve the modelling used in dissemination and implementation research. Toward that end, we thought to share best principles of systems science that we have successfully applied in our own studies toward enhancing implementation science. Best principles, as distinct from the more customary term best practices, are used to underscore the need to extract the core issues from the context in which they are embedded in order to better ensure that they are transferable across settings [27]. For a full treatment of the principles, meaning fundamental truths, of systems science, see the recent text by Mobus and Kalton [28].

## The Modelling Process

The problem we were attempting to solve in our pilot study was to improve primary care screening and care coordination at chairside, meaning in a dental setting rather than a medical or other setting [19]. While we had both championed and been involved in previous initiatives that integrated oral health and primary care [29–32], our idea was to support dental hygienists in practicing to the full extent of their training so that they might effectively implement evidence-based guidelines for tobacco use, hypertension and diabetes screening, and nutrition counselling in dental settings [33]. We are principally focused on advancing health equity and ensuring that population groups who lack oral health and primary care are linked to accessible providers and care settings in their own communities, whenever possible [7, 30].

**Fig. 1:**
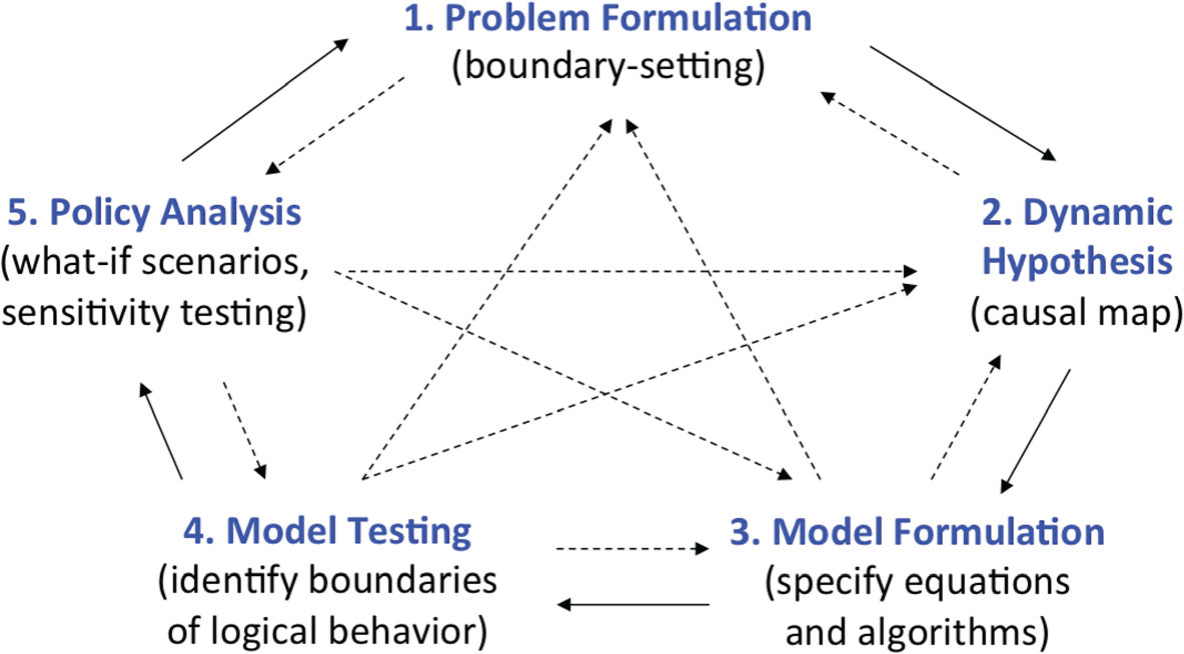
Stages of the modelling process. The modelling process depicted as an iterative sequence of steps beginning with problem definition and concluding with policy analysis

The modelling process is depicted in Fig. 1 as an iterative sequence of steps beginning with problem definition and concluding with policy analysis. Importantly, insights are acquired at all stages of the modelling process. While Fig. 1 illustrates a return to problem definition upon completion of a modelling project, Sterman [34] emphasises that it may also be appropriate to iterate within the process for the purpose at hand, returning to previous steps or anticipating scenarios ahead of time.

Next, we propose and illustrate four best principles derived from our ongoing systems science research and scholarship that may guide, and perhaps even motivate, implementation scientists in their own studies and thinking. The overarching theme of these best principles involves meaningfully informing the modelling process. It is our belief that this aspect of dissemination and implementation research demands concerted attention in order to meaningfully grapple with the complex and challenging problems faced in adopting and integrating evidence-based health interventions and changing practice patterns within specific settings [31].

### Best Principle #1: Model the problem, not the system

Sterman rightly deserves credit for driving home the importance of modelling the problem, not the system [34]. Accordingly, we began our aforementioned pilot study by conducting formative research about the views of dental providers (both dental hygienists and dentists) on primary care coordination at chairside [20]. Findings were that both the dental hygienists and dentists interviewed as part of this research failed to use evidence-based guidelines to screen their patients for primary care-sensitive conditions such as hypertension and diabetes [20]. Nonetheless, all of the participating dental hygienists and dentists reported using electronic devices at chairside to obtain web-based health information in caring for their patients [20]. Hence, we worked collaboratively to develop a clinical decision support system for use by dental hygienists to support them in providing patient care at the level of their full scope of practice [19, 33].

**Fig. 2:**
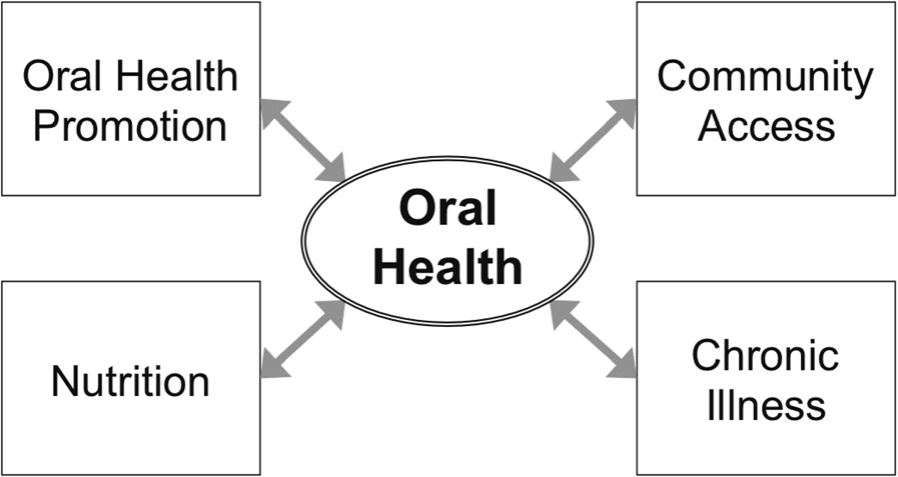
Conceptual model focused on the problem of oral health. A conceptual model that identifies the key problem variable of a systems science study as oral health, shown as influencing and influenced by distinct factors at the individual and community scales

Formerly, we developed a causal map to understand the complex set of causal pathways that are involved and the time delays that accrue over a life course toward developing effective oral health interventions for older adults [5]. A simplified version of this conceptual model is presented below, identifying the key problem variable of our systems science study as “oral health,” shown as influencing and influenced by distinct factors at the individual and community scales (Fig. 2). At the individual scale are factors such as nutrition and the presence of chronic illness. Individuals intersect with the community scale in terms of factors such as exposure to oral health promotion interventions and community access to health screening and healthcare.

In subsequent research, we reframed the locus of concern around health equity more broadly, requiring us to reconsider how an individual’s health status reflects a broader distribution of social and health disparities that vary by population subgroups. An orientation toward health equity warrants a broader model conceptualisation than health per se [35].

Attempts to model the system rather than the problem are bound to lead to confusion and futility [34]. Our training and experience in systems science directed us away from trying to design an integrated system of oral and primary care and focused our attention instead on supporting dental hygienists to adhere to evidence-based tobacco use, hypertension and diabetes screening, and nutrition counselling referral guidelines [19, 20, 33]. Formative research and interdisciplinary collaboration are invaluable in steering implementation scientists toward modelling the problem, not the system.

### Best Principle #2: Pay attention to what is important, not just what is quantifiable

Meadows was a rigorous systems scientist who inspired her colleagues and students to pay attention to what is important – be it justice, democracy, security, freedom, truth, or love – even if it cannot be precisely defined or measured [36]. Unfortunately, despite the critical importance of qualitative information, certain researchers restrict the constructs and variables in their models to those for which numerical data are available, and include only those parameters that can be estimated statistically [37]. Yet, in a comprehensive article on collecting and analysing qualitative data for system dynamics [38], Luna-Reyes and Andersen argue convincingly that qualitative data and their analysis also have a central role to play at all stages of the modelling process. Using strategies such as theirs, qualitative statements can be used to derive causal relationships.

**Fig. 3:**
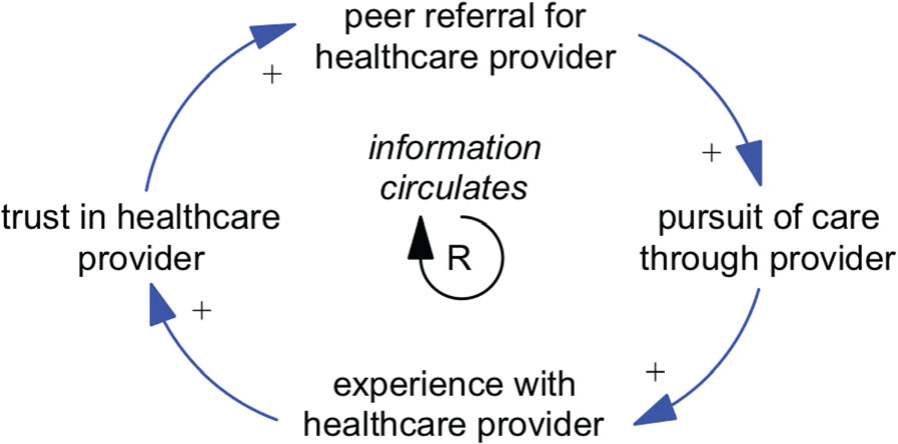
Example of causal mapping from qualitative data. A reinforcing loop reflecting the essence of a comment (qualitative data) from a focus group participant regarding the importance of a peer network in recommending healthcare providers

As an example, in a Spanish-language focus group about dental care conducted with men aged 50 years and older who reside in northern Manhattan, New York, and had immigrated from the Dominican Republic, one participant explained: “*Sometimes you* [go to the dentist] *because you get a referral from a friend: ‘Oh, so and so. Now that’s a good dentist.’ So you go, more or less, because of that reference. It’s not like you go* [because of] *where it is, but because you had a referral, and that information circulates.*” This explanation summarises the importance of the peer network in recommending healthcare providers. A reinforcing loop reflecting the essence of this comment is depicted in Fig. 3. The notion that information circulates points to the mechanism by which an individual’s experience with a provider translates into referrals or recommendations for the provider, inducing her or his social ties to then pursue care with the recommended provider. An intermediate construct of trust in healthcare provider extends beyond the direct comment but helps to articulate the basis of the recommendation.

Because dissemination and implementation studies are based on the mechanisms through which health information, interventions and evidence-based clinical practices are adopted in public health, community and healthcare service use in a variety of settings, a broad range of methodological approaches are employed [39]. These include both traditional designs, such as randomised controlled trials, and newer approaches such as hybrid effectiveness-implementation designs [40, 41]. While mixed methods approaches are endorsed in implementation science, there is a need for greater attention to connectedness across program levels and components [40].

We are at the point in our implementation science study of primary care coordination by dental hygienists at chairside where we need to create a causal map (also known as causal loop diagram) to provide a systematic way to develop dynamic hypotheses and identify important feedback loops [42]. In a causal map, it is possible to ascribe certain variables to specific scales, e.g. community, interpersonal and individual. Because systems science models are not limited to constructs that are precisely defined or measured, deep thinking and multiple perspectives may help guide implementation scientists to pay attention to what is important, not just what is quantifiable.

### Best Principle #3: Leverage the utility of models as boundary objects

According to Black, a boundary object is “*a representation—perhaps a diagram, sketch, sparse text, or prototype—that helps individuals collaborate effectively across some boundary, often a difference in knowledge, training, or objective*” ([43], p. 76). For research teams such as ours, whose members possess expertise in diverse domains, boundary objects are useful for coordinating knowledge and objectives and for developing a shared vocabulary about the problem to be solved collaboratively [44].

**Fig. 4:**
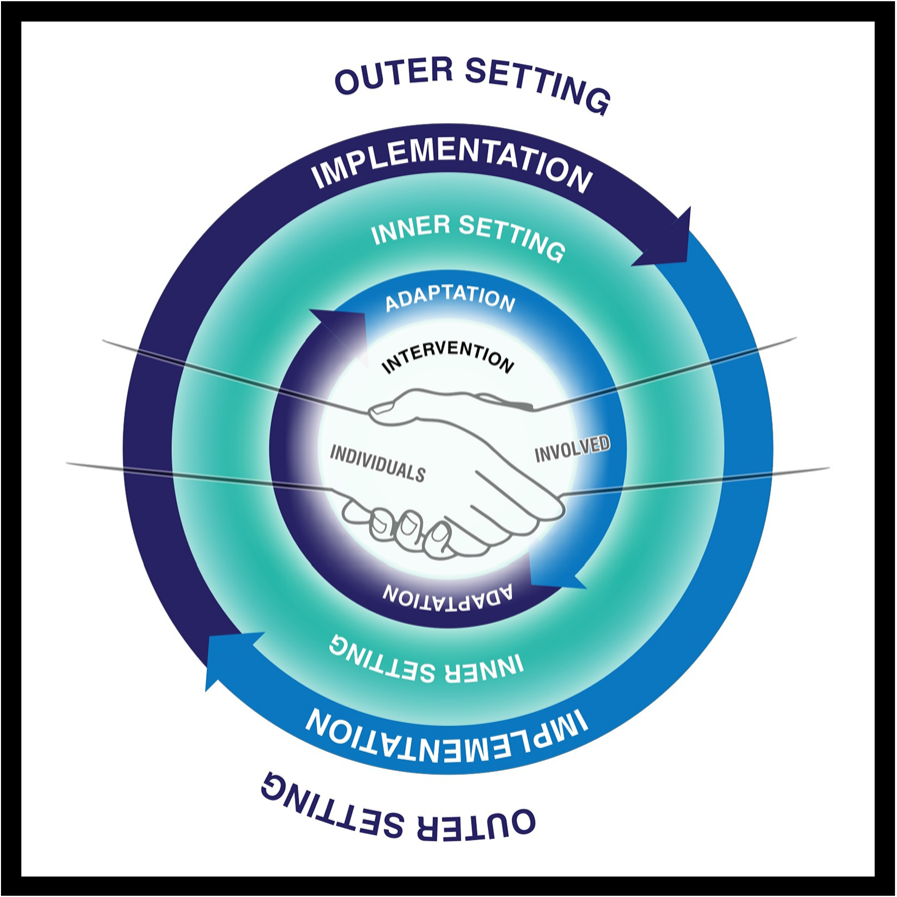
Simplified model of the Consolidated Framework for Implementation Research. The five major domains of the Consolidated Framework for Implementation Research (the intervention, the inner and outer settings, the individuals involved, and the process by which implementation is accomplished) are represented in this simplified model, along with the process of adaptation

The conceptual framework that informs our interventions is the Consolidated Framework for Implementation Research (CFIR) [45]. While this proved to be incredibly helpful to us in designing and evaluating our implementation science pilot study, we found the accompanying graphic to be difficult to understand. Hence, we developed a simplified model that was derived from previous examples used in our systems science research. As shown in Fig. 4, the five major domains of the CFIR (the intervention, the inner setting, the outer setting, the individuals involved and the process by which implementation is accomplished) are represented in the simplified model, along with the process of adaptation [20].

This graphic proved to be both intuitive and accessible to our interdisciplinary team members, so much so that we have created project-specific models for a series of papers [19, 20, 33]. We now consider our CFIR model to be a boundary object that facilitates team collaboration.

Note that, from a modelling perspective, a boundary object is “*a socially constructed artefact for building trust and agreement*” ([46], p. 4, citing [47]). For boundary objects to be useful, they must be modifiable and readily perceptible representations that embody the dependencies among resources and goals of team members [48]. While boundary objects represent local knowledge, they may be shared across networks and thus play a significant role in creating synergies which in turn sustain local initiatives [49]. Developed models used as boundary objects may benefit implementation scientists through building trust and agreement that represent local knowledge.

### Best Principle #4: Adopt a portfolio approach to model building

As we alluded to at the outset of this paper, our research team led by the authors – an implementation scientist (MEN) and a systems scientist (SSM) – has developed a portfolio of conceptual, statistical, spatial and simulation models that utilise the multiple information streams associated with our research projects [44]. A chief advantage of the portfolio approach in a collaborative research context is that it provides multiple entry points and checkpoints to the modelling process, facilitating input from different team members [6]. A further benefit is that team members often work in parallel to develop separate but related models in diverse ways, exploring the simulated consequences of alternative assumptions [6].

For instance, in our ongoing project, Integrating Social and Systems Science Approaches to Promote Oral Health Equity, our modelling team has gained important insights by adopting a portfolio approach that incorporates different methods of systems science, including system dynamics, agent-based modelling, geographic information science and social network simulation, in models that help to explore challenges to realising oral health equity for older adults [6, 35]. This portfolio approach to systems science modelling enables our research team to interpret and triangulate between different scenarios at distinct geographic and temporal scales. An inventory of the simulation models in our portfolio that highlights their links to other models in the portfolio is provided in Additional file 1.

In essence, then, the construction of a portfolio of models confers flexibility to the modelling process and is especially conducive to collaboration, allowing for multiple opportunities for input and adjustment of models by different members of the research team. Further, the portfolio approach leverages the iterative nature of the modelling process and encourages exploration with ‘flawed’ models rather than aiming for perfection with ‘kitchen sink’ models. Implementation scientists may profit from adopting a portfolio approach to model building that confers flexibility and is conducive to collaboration.

## Conclusions

**Fig. 5:**
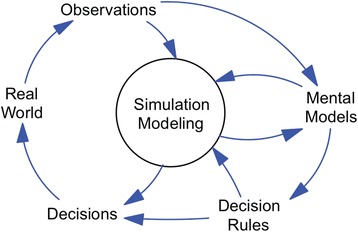
Simulation modelling in context. The practice of simulation modelling is situated amidst an ongoing process of observing the real world, formulating mental models of how it works, setting decision rules to guide behaviour, and from these heuristics, making decisions that in turn affect the state of the real world

In order to improve our mental models of the real world, system scientists have developed and leveraged methods such as system dynamics, agent-based modelling, geographic information science and social network simulation. As articulated by Sterman [34] (Fig. 5), the practice of simulation modelling is situated amidst an ongoing process of observing the real world, formulating mental models of how it works, setting decision rules to guide behaviour, and from these heuristics, making decisions that in turn affect the state of the real world. Simulation modelling offers a mechanism for what Sterman calls ‘double-loop learning’ [34], arriving at insight from the process of virtual experimentation afforded by simulation modelling, in addition to learning from experiences in the real world. The two-way relationship between mental models and simulation modelling underscores the essential nature of learning through the modelling process.

Because as humans we can only process a limited amount of information in our heads as ‘thought experiments’, we need to simulate computer models to transcend our mental models. In short, to understand dynamic complexity, we need the ability to simulate. Otherwise, our understanding will be limited.

Modelling, then, is the art and science of linking system structure to behaviour for the purpose of changing structure to improve behaviour. A useful computer model creates a knowledge repository and a virtual library for internally consistent exploration of alternative assumptions. Among the benefits of systems modelling are iterative practice, participatory potential and possibility thinking.

We trust that the best principles proposed here will resonate with our fellow implementation scientists and that applying them to the modelling process will abet the translation of research into effective policy and practice. Table 1 provides a summary of the four best principles discussed herein for informing the modelling process, along with recommendations for action by implementation scientists and the contributing thought leaders whose references we cited.

**Table 1:** Summary of best principles from systems science for informing the modelling process, recommendations for action by implementation scientists and contributing thought leaders and key references

Best principle	Recommendations	Thought leader [Reference]
1. Model the problem, not the system	Conduct formative research; construct models collaboratively in interdisciplinary teams	Sterman [34]
2. Pay attention to what is important, not just what is quantifiable	Use qualitative data to derive causal relationships; be guided by deep thinking and multiple perspectives	Meadows [36]
3. Leverage the utility of models as boundary objects	Create modifiable and readily perceptible representations of models; build trust and agreement by representing local knowledge	Black [43]
4. Adopt a portfolio approach to model building	Work in parallel to develop separate but related models in diverse ways; encourage exploration with ‘flawed’ models rather than aiming for perfection	Metcalf [6]

As Sterman cautions us, “*What prevents us from overcoming policy resistance is not a lack of resources, technical knowledge, or a genuine commitment to change. What thwarts us is our lack of a meaningful systems thinking capability*” ([9], p. 513).

## Additional file

Additional file 1: Summary of simulation models in systems science portfolio. (DOCX 22 kb)
