# Supplementary material for: Enhancing implementation science by applying best principles of systems science
Source: Health Res Policy Syst. 2016 Oct 4;14:74. doi: 10.1186/s12961-016-0146-8 (PMC5050576; doi:10.1186/s12961-016-0146-8)
Supplement: Additional file 1: — Summary of simulation models in systems science portfolio. (DOCX 22 kb) [file 12961_2016_146_MOESM1_ESM.docx]

Additional file 1. Summary of Simulation Models in Systems Science Portfolio

| Model | Model Description | Links to Other Models in Portfolio |
| --- | --- | --- |
| 1. Model A-SD | Stock-flow system dynamics (SD) model of social diffusion of care-seeking behavior | Compares with Model A-ABM |
| 2. Model A-ABM | Agent-based model (ABM) of diffusion through equivalent states as Model A-SD | Compares with Model A-SD |
| 3. Model B | SD model of preventive screening as path to treatment, accounting for capacity constraints and perception delays. | Conceptual extension of  Model A-SD |
| 4. Model C | ABM of preventive screening as path to treatment, includes care facilities and older adult agents interacting in GIS environment. | Integrates aspects of Model B and Model A-ABM |
| 5. Model C-1 | Streamlined version of Model C used to test effectiveness of alternative schedules for preventive screening. | Derivative of  Model C |
| 6. Model D | ABM of preventive screening and diffusion of care-seeking behavior in a GIS environment. | Incorporates elements from Model C |
| 7. Model E | An ABM of social influence on transport choices in mediating access to care in a GIS environment. | Incorporates elements from Model D |
| 8. Model F | An ABM designed to engage our collaborative research team in group model-building that simulates dynamic networks forming from social activities. |  |
| 9. Model G | An ABM designed to simulate social capital using dynamic networks. | Network dynamics from Model F |
| 10. Model H | An ABM designed to incorporate the “best pieces” of other models from our portfolio. |  |
| 11. Model I | An ABM of transport choices accounting for GIS-based road transport networks that includes carpooling vans. | Conceptual extension of  Model E |

**Causal Maps in Model Portfolio:**

1. A systems perspective motivating our original research design, corresponding with the domains articulated in Figure 1, as presented in Metcalf et al., 2011.

2. A collaboratively developed boundary object during our second group model-building workshop using physical materials.

3. Conceptual framework emphasizing impact of expanded Medicaid insurance coverage on access to care, quality of care, and oral health equity.

4. Framework for dynamic network model that identifies elements of social capital in the system that influence health equity (Figure 1 in Wang et al., 2016).

5. Causal maps developed at Group Model Building (GMB) workshop 3 using Connection Circles to explore dimensions of accessibility.

6. Causal map modified at GMB workshop 4 illustrating method of translation from qualitative data to inferences about causal relationships.

**References to models in our portfolio:**

(Models A-C):

Metcalf SS, Northridge ME, Widener MJ, Chakraborty B, Marshall SE, Lamster IB. Modeling social dimensions of oral health among older adults in urban environments. Health Educ Behav. 2013;40 Suppl 1:63S-73S.

(Model B):

Metcalf SS, Widener MJ, Northridge ME, Paich M, Marshall S, Lamster IB. 2011. Modeling the dynamics of dental health among older adults. Proceedings of the 29^th^ International Conference of the System Dynamics Society. Washington, DC: System Dynamics Society; 2011. <http://www.systemdynamics.org/conferences/2011/proceed/papers/P1307.pdf>. Accessed 15 Jun 2016.

(Model C-1):

Widener MJ, Northridge ME, Wang P, Kum SS, Chen Z, Marshall SE, Metcalf SS. Using agent-based modeling to develop strategies for maximizing participation in an oral health screening program for older adults in northern Manhattan. Lansing, MI: 15^th^ International Symposium in Medical/Health Geography; 2013.

(Model D):

Metcalf S, Wang H, Kum S, Jin Z, Wang P, Widener M, Kunzel C, Marshall S, Northridge M. Modeling social factors of oral health equity for older adults. In Pasupathy R, Kim S, Tolk A, Hill R, Kuhl M. (eds.), Proceedings of the 2013 Winter Simulation Conference, 3994-3995. Washington, DC: Informs Simulation Society; 2013 <http://informs-sim.org/wsc13papers/includes/files/389.pdf>. Accessed 15 Jun 2016.

(Model E):

Jin Z. Influence of social support and transportation on older adults’ access to oral healthcare. MS Thesis, University at Buffalo Department of Geography; 2014.

Jin Z, Northridge M, Wang H, Kunzel C, Kum S, Metcalf S. The influence of social support and transportation on older adults’ access to oral healthcare. Chicago, IL: 111^th^ Annual Meeting of the Association of American Geographers; 2015.

(Model G):

Wang H, Northridge ME, Kunzel C, Zhang Q, Kum SS, Gilbert JL, Jin Z, Metcalf SS. Modeling social capital as dynamic networks to promote access to oral healthcare. Lecture Notes in Computer Science Volume 9708, Chapter 12. Washington, DC: International Conference on Social Computing, Behavioral-Cultural Modeling & Prediction and Behavior Representation in Modeling and Simulation (SBP-BRiMS); 2016. p. 117-130.

(Model H):

Jin Z, Gilbert J, Kum S, Zhang Q, Wang H, Kunzel C, Northridge M, Metcalf S. Modeling multi-scalar factors of oral health equity for older adults. San Francisco, CA: 112^th^ Annual Meeting of the Association of American Geographers; 2016.

(Model I):

Zhang Q, Metcalf S. Modeling the accessibility of healthcare facilities to older adults in urban areas. San Francisco, CA: 112^th^ Annual Meeting of the Association of American Geographers; 2016.

Zhang Q. Modeling the accessibility of oral healthcare facilities for older adults. MS Thesis, University at Buffalo Department of Geography; 2016.

**References to causal maps in our portfolio:**

(Causal Map 1)

Metcalf SS, Northridge ME, Lamster IB. A systems perspective for dental health in older adults. Am J Public Health. 2011;101(10):1820-1823.

(Causal Map 2)

Kum SS, Wang H, Jin Z, Xu WW, Mark J, Northridge ME, Kunzel C, Marshall SE, Metcalf SS. 2015. Boundary objects for group model building to explore oral health equity. Proceedings of the 33rd International Conference of the System Dynamics Society. Cambridge, MA: System Dynamics Society; 2015. <http://www.systemdynamics.org/conferences/2015/proceed/papers/P1302.pdf>. Accessed 16 Jun 2016.

(Causal Map 3)

Metcalf SS, Birenz SS, Kunzel C, Wang H, Schrimshaw EW, Marshall SE, Northridge ME. The impact of Medicaid expansion on oral health equity for older adults: a systems perspective. J Calif Dent Assoc. 2015;43(7):369-377.
